# Establishment of a Lung Cancer Discriminative Model Based on an Optimized Support Vector Machine Algorithm and Study of Key Targets of Wogonin in Lung Cancer

**DOI:** 10.3389/fphar.2021.728937

**Published:** 2021-09-22

**Authors:** Lin Wang, Jianhua Zhang, Guoyong Shan, Junting Liang, Wenwen Jin, Yingyue Li, Fangchu Su, Yanhua Ba, Xifeng Tian, Xiaoyan Sun, Dayong Zhang, Weihua Zhang, Chuan liang Chen

**Affiliations:** ^1^ Department of Radiotherapy, People’s Hospital of Zhengzhou, Zhengzhou, China; ^2^ Medical Engineering Technology and Data Mining Institute, Zhengzhou University, Zhengzhou, China; ^3^ Clinical Bioinformatics Experimental Center, Henan Provincial People’s Hospital, People’s Hospital of Zhengzhou University, Zhengzhou, China

**Keywords:** lung cancer, wogonin, serum index, SVM, network pharmacology, key targets

## Abstract

An optimized support vector machine model was used to construct a lung cancer diagnosis model based on serological indicators, and a molecular regulation model of Wogonin, a component of *Scutellaria baicalensis*, was established. Serological indexes of patients were collected, the grid search method was used to identify the optimal penalty coefficient C and parameter g of the support vector machine model, and the benign and malignant auxiliary diagnosis model of isolated pulmonary nodules based on serological indicators was established. The regulatory network and key targets of Wogonin in lung cancer were analyzed by network pharmacology, and key targets were detected by western blot. The relationship between serological susceptibility genes and key targets of Wogonin was established, and the signaling pathway of Wogonin regulating lung cancer was constructed. After support vector machine parameter optimization (*C* = 90.597, *g* = 32), the accuracy of the model was 90.8333%, with nine false positives and two false negative cases. Ontology functional analysis of 67 common genes between Wogonin targets and lung cancer–related genes showed that the targets were associated with biological processes involved in peptidye-serine modification and regulation of protein kinase B signaling; cell components in the membrane raft and chromosomal region; and molecular function in protein serine/threonine kinase activity and heme binding. Kyoto Encyclopedia of Genes and Genomes analysis showed that the regulation pathways involved the PI3K-Akt signaling pathway, ERBB signaling pathway, and EGFR tyrosine kinase inhibitor resistance. *In vitro* analyses using lung cancer cells showed that Wogonin led to significantly increased levels of cleaved caspase-3 and Bad and significantly decreased Bcl-2 expression in a concentration-dependent manner. ErbB4 expression also significantly decreased in lung cancer cells after treatment with Wogonin. A regulatory network of Wogonin regulating lung cancer cell apoptosis was constructed, including the participation of serological susceptibility genes. There is a certain regulatory effect between the serological indexes that can be used in the diagnosis of lung cancer and the key targets of Chinese herbal medicine treatment of lung cancer, which provides a new idea for the diagnosis, treatment and prognosis of clinical lung cancer.

## 1 Introduction

Lung cancer has the highest mortality rate among all cancers worldwide (Schwartz and Cote, 2016). China has the largest number of lung cancer patients in the world, and the incidence rate continues to rise. Lung cancer thus represents a serious threat to the health of Chinese citizens (Nasim et al., 2019). Early diagnosis of lung cancer can significantly improve the 5-years survival rate of patients (Weller, 2021). Many methods for the diagnosis of lung cancer are currently available, such as imaging, bronchial ultrasound examination and exhaled air examination (Ar Me Nteros et al., 2019), but these are not widely used for the early screening of lung cancer. Several tumor markers for lung cancer have been identified and applied in the clinical diagnosis of lung cancer, such as neuron-specific enolase (NSE), glycoprotein antigen 153 (CA153), cytokeratin 19 fragment (CYFRA21-1) and glycoprotein antigen 125 (CA125) (Wang J. et al., 2018; Kang et al., 2019). However, an effective index to independently detect lung cancer at early stages has not been found.

The main treatment for lung cancer is chemotherapy, but the success rate is not high (Hirsch et al., 2017; Jones and Baldwin, 2018). Therefore, more effective treatments for lung cancer are required. Chinese herbal medicine is characterized as containing multiple components with multiple therapeutic objectives and a comprehensive regulation for treatment. The application of Chinese herbal medicine in the treatment of lung cancer has certain advantages in stabilizing lesions, extending patient life, and improving patient quality of life (Liao et al., 2017; Xiang et al., 2017; Tang et al., 2019). The Chinese herbal medicine *Scutellaria baicalensis* is the dry root of the herb *Scutellaria labiatae*. According to Shennong’s Herbal scripture in historical records, *S. baicalensis* has the functions of being bitter, cold, hot and wet, detoxifying fire and stopping bleeding (Wang Z. L. et al., 2018; Zhao et al., 2019). Recent pharmacological studies on *S. baicalensis* have shown that it has antibacterial, anti-pathogen, antioxidant, immunologic regulation, anti-tumor, antipyretic, sedative, and analgesic activities (Chen et al., 2016; Dinda et al., 2017; Cheng et al., 2018). Wogonin, a natural flavonoid, is a volatile component of *S. baicalensis* with a variety of pharmacological activities, such as anti-inflammatory, antiviral, and antioxidant activities. In recent years, its anti-tumor and immunomodulatory effects have been widely reported (Wang et al., 2021). Several studies have investigated the treatment of lung cancer with *S. baicalensis* components, including Wogonin, Baicalin, Baicalein and total scutellaria flavone (Chen et al., 2013; Yang and Sun, 2016; Xu et al., 2017). However, overall systematic research on the treatment of lung cancer by the volatile components of *S. baicalensis* is still lacking.

Support vector machine (SVM) is a machine learning algorithm based on the principle of structural risk minimization that can predict the results of a small sample of data (Parseei and Stashuk, 2012; Chang et al., 2013). SVM has thus been widely studied and applied in the medical field. Identifying optimized parameters leads to more accurate simulations. In the construction of a SVM classifier, the most important step is finding the appropriate penalty coefficient C and parameter g. The function of penalty parameter C is to adjust the misclassification tolerance; g is an important parameter of RBF(radial basis function) kernel function. The different choice of g will directly lead to the change of SVM classification accuracy. Both penalty parameter C and RBF kernel function g directly affect the classification effect of SVM.

The aim of our investigation was to provide a theoretical basis for the treatment of lung cancer with volatile extractives of *S. baicalensis*. Combined with the serological indicators of lung cancer patients, we established a diagnostic model of benign and malignant pulmonary nodules through the SVM model and optimized the SVM parameters C and g using the grid search method to improve the accuracy of the model. Combined with network pharmacology, the key targets and regulatory pathways of the Wogonin in the intervention of lung cancer were analyzed. We also conducted *in vitro* experiments to explore the main mechanisms of the action of the key volatile components on lung cancer. The correlation between Wogonin target and lung cancer serological indicators was analyzed, and the regulatory network of the interactions was constructed. Our findings provide new research ideas for the diagnosis and treatment of clinical lung cancer.

## 2 Materials and Methods

### 2.1 General Information

A total of 600 patients, including 300 patients with lung cancer and 300 patients with benign pulmonary nodules, were enrolled from July 2017 to December 2020. Among the 300 patients with lung cancer, there were 162 males and 138 females; the average patient age was 60.4 ± 9.8 years. Among the 300 patients with benign pulmonary nodules, there were 200 males and 100 females; the average patient age was 56.3 ± 13.2 years. All patient cases were confirmed by pathology.

### 2.2 Detection of Serum Tumor Markers

Venous blood (2 ml) was taken from all subjects on an empty stomach at 8:00 am. The samples were left standing for 1 h and centrifuged at 2000 rpm for 5 min. The serum was then removed for analysis. Four serological markers were detected in each serum sample: CA153, CYFRA21-1, CA125 and NSE. Radioimmunoassay was used to detect NSE, ELISA was used to detect CYFRA21-1, and Roche immunoanalyzer was used to detect CA153 and CA125. All the tests were conducted in strict accordance with the kit instructions.

### 2.3 SVM Model

The 600 patients were divided into two groups: the training set consisted of 480 patients (240 lung cancer cases and 240 benign cases) and the test set consisted of 120 patients (60 lung cancer cases and 60 benign cases). MATLAB R 2016A was used to build the SVM model, and the kernel function was set as RBF kernel function; Eq. 1 is shown below.

$$K\left({\text{x}}_{i},x_{j}\right)=\exp\left(-g{\left\|x_{i}-x_{j}\right\|}^{2}\right)$$ (1)

We used the grid search method to identify the optimal penalty factor C and parameter g. The grid search method is an exhaustive search method for specifying parameter values. It obtains the optimal learning algorithm by optimizing the parameters of the estimated function through cross validation. The grid search method is used to determine the optimal parameters of the kernel function. The test steps are as follows:

** **Begin.
1) Set the range of c and g values and search step size of grid search. Here, we set it as *g* = 2^−8^–2^8^ and the step size is 0.5; *c* = 2^−8^–2^8^, step size 0.5.
2) Divide the data set into 10 subsets of the same size (5 subsets for both positive and negative samples). The current values of c and g were used for cross-effective calculation, and the accuracy of the cross-effective prediction was recorded.
3) Repeat step 2 until the grid search is completed.
4) Draw the contour map of prediction accuracy, and select the optimal parameter value accordingly. Finally, the accuracy contour chart is obtained.
** **End.

### 2.4 Mining of Susceptibility Genes Corresponding to Serological Indicators

The four serological indexes CA153, CYFRA21-1, CA125 and NSE were screened for susceptibility genes by literature retrieval and mining techniques.

### 2.5 Network Pharmacological Analysis

#### 2.5.1 Screening and Identifying the Volatile Active Components of *S. Baicalensis*

The volatile components of *S. baicalensis* were extracted by gas chromatography–mass spectrometry (GC-MS) (Zhu et al., 2021). Volatile components of *S. baicalensis* were screened using the Traditional Chinese Medicine Systems Pharmacology (TCMSP) database under conditions of an oral bioavailability score ≥30% and druggability score ≥0.18. We downloaded the chemical structure files corresponding to the active components.

#### 2.5.2 Target Prediction of the Active Ingredients of *S. Baicalensis* and Lung Cancer

We determined the specific chemical information for Wogonin using the PubChem database. We predicted the target genes using BATMAN-TCM combined with the web-based SwissTargetPrediction tool.

We searched the NCBI gene and GeneCards databases using the keyword “lung cancer” and obtained target gene–related data for lung cancer regulation. We used the UniProt databases to improve the target’s UniProt ID and gene name.

The overlapping targets of TCM and disease targets were screened by Venn diagram.

#### 2.5.3 Enrichment Analysis and Protein-Protein Interaction Analysis

We entered the overlapping target genes into the Cytoscape software and used GO and KEGG in the ClueGo plug-in for Gene Ontology (GO) and Kyoto Encyclopedia of Genes and Genomes (KEGG) enrichment analysis of the Chinese herbal medicine volatile extractives acting on lung cancer–related targets. The parameters were set as ClueGo:Function, GO and KEGG and Use GO Term Fusion (*p* ≤ 0.05).

The overlapping genes were considered as hub genes and analyzed using online STRING (https://www.string-db.org/) to obtain the PPI, with the species limited to “Homo sapiens” and a confidence score >0.990. The TSV format file, which was downloaded from the STRING database, was imported into Cytoscape3.7.1 for analysis. Cytoscape 3.7.1, which is widely applied in network pharmacology research, was used to construct and visualize the PPI network.

### 2.6 Experimental Verification

#### 2.6.1 Cell Culture and Treatments

The human lung cancer cell line A549 was maintained in special medium at 37°C under an atmosphere of 5% CO_2_. Cells were treated with 0, 5, 15, or 20 μmol/L Wogonin for 24 h and then subjected to experimental analyses; the concentrations of Wogonin were established based on a previous study (Wang and Cui, 2019).

#### 2.6.2 Western Blot

Cells were lysed and protein quantification was performed using a kit (Wanleibio Co., Ltd., Shenyang, China). Protein lysates (20 μg) were separated by SDS-PAGE and transferred to a polyvinylidene fluoride membrane (Millipore, Bedford, MA, United States). The membrane was washed in Tris buffered saline with 0.5% Tween 20 (TBST) for 5 min and then blocked for 1 h in 5% skimmed milk. The membranes were incubated with antibodies against caspase-3, cleaved caspase-3, BAD, Bcl-2, and β-actin (1:2000 dilution; Wanleibio Co., Ltd.) overnight at 4°C. The membranes were washed three times with TBST for 5 min each and then incubated for 1 h at 37°C with rabbit HRP-conjugated anti-mouse antibody (1:1,000) (Wanleibio Co., Ltd.), followed by three washes with TBST for 5 min each. Protein bands were visualized using electrochemiluminescence reagent (Wanleibio Co., Ltd.). β-actin served as an internal control to normalize the band density.

### 2.7 Statistical Analyses

All experimental data were tested for homogeneity and normality and are expressed as mean ± standard deviation. Analyses were performed using SPSS23.0 software. For measurement of data, *t*-test was used for comparison between two groups. *p* < 0.05 was considered as statistically significant.

## 3 Results

### 3.1 Pathological Sections of Benign and Malignant Lung Cancer

**FIGURE 1:**
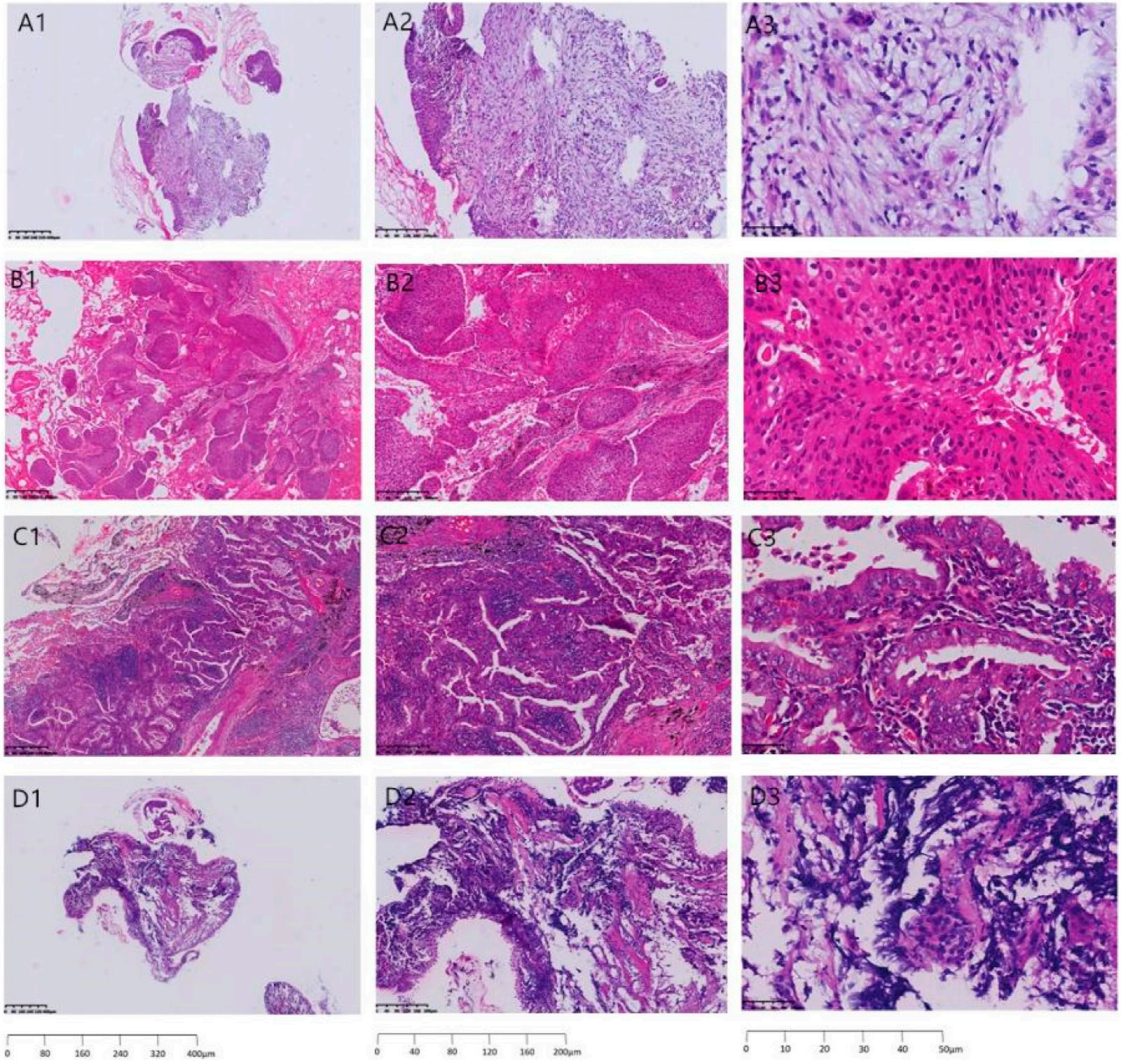
Pathological section of benign and malignant lung cancer. **(A)** benign nodules, **(B)** squamous cell carcinoma of the lung, **(C)** adenocarcinoma of the lung, and **(D)** small cell lung cancer; A1,B1,C1,D1) 40× under the microscope, A2,B2,C2,D2)100× under the microscope, A3,B3,C3,D3) 400× under the microscope.

Figure 1 shows the pathological sections of benign and malignant lung cancers, in which A, B, C and D represent the pathological sections of benign nodules, squamous cell carcinoma of the lung, adenocarcinoma of the lung, and small cell lung cancer, respectively.

### 3.2 Differential Analysis of Serum Markers

Serum concentrations of lung cancer markers NSE, CYFRA21-1, CA125, and CA153 were all significantly higher in patients with lung cancer than those in the benign group (Table 1).

**TABLE 1:** Concentrations of four serum tumor markers.

Serum tumor markers	Benign SPNs group (*n* = 300)	Malignant SPNs group (*n* = 300)	Z	*p*
NSE (ng/ml)	12.11 (11.49, 12.59)	14.83 (12.56, 16.17)	−9.896	<0.001
CYFRA21-1 (ng/ml)	2.16 (1.99, 2.30)	6.77 (4.75, 7.84)	−17.627	<0.001
CA125 (U/mL)	18.42 (14.95, 21.08)	46.30 (36.05, 54.05)	−16.653	<0.001
CA153 (U/mL)	10.37 (9.39, 11.33)	17.77 (14.49, 19.64)	−13.258	<0.001

### 3.3 Training and Test Results of the SVM Model

**FIGURE 2:**
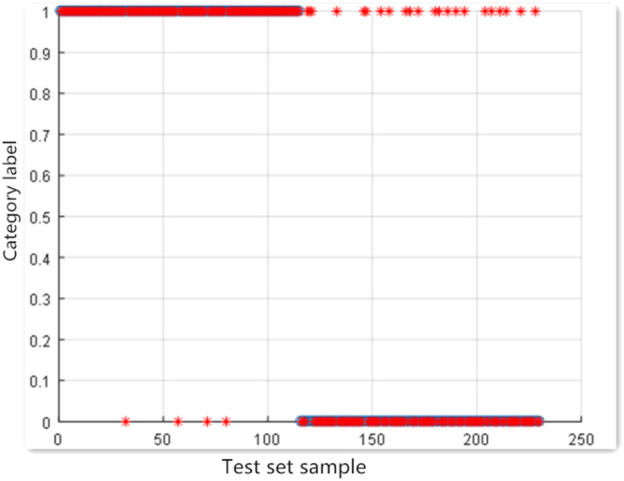
SVM test results with default parameters.

The four serological indexes NSE, CYFRA21-1, CA125 and CA153 were modeled under the default parameters (*C* = 1, *g* = 0.5); the accuracy of the test set was 87.5%, with 13 false positives and 2 false negatives. The results are shown in Figure 2.

**FIGURE 3:**
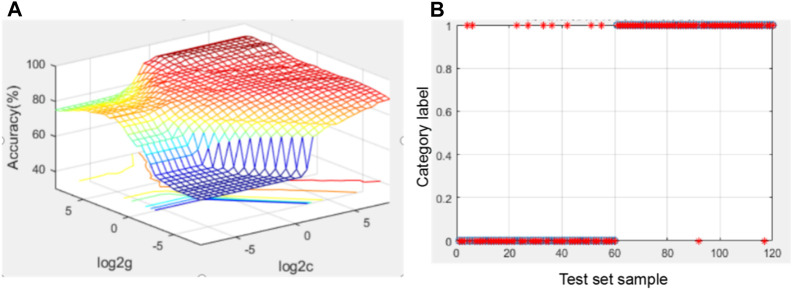
Diagram of optimization results using the grid search method and SVM test results using the four indicators. **(A)** Optimization result graph of the grid searching method; for the 3D view, the group C and g with the highest accuracy was selected as the optimal C and g; **(B)** graph of SVM test results under optimal parameters.

Searching for the optimal parameters using grid search method led to the following parameters: *C* = 90.597, *g* = 32. Under these parameters, the accuracy of test set was 90.8333%; the number of false positives was 9 and the number of false negatives was 2. C, g step size is 0.5. The results are shown in Figure 3.

### 3.4 Mining of Susceptibility Genes Associated With Four Lung Cancer Serum Markers

Through literature retrieval and mining technology, we obtained susceptibility genes corresponding to the four serological indexes associated with lung cancer as follows: NSE, *LATS1*; CA153, *BCAR4*; CYFRA21-1, YAP; and CA125, *PVT1*.

### 3.5 Results of Network Pharmacological Analysis

#### 3.5.1 Identification of Volatile Components of *S. Baicalensis*

**FIGURE 4:**
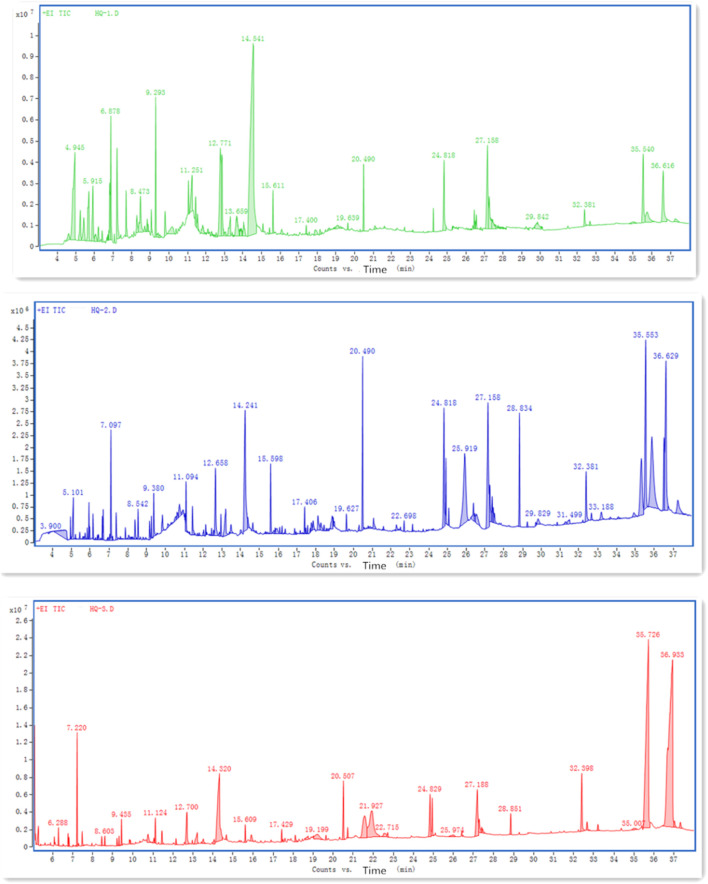
Ion flow diagram of the three extraction methods of *S. baicalensis* detected by GC-MS. **(A)** Determination of ion flow chart by GC-MS for *S. baicalensis* extracted by ethanol; **(B)** GC-MS detection of ion flow diagram of *S. baicalensis* extracted by methanol; **(C)** GC-MS detection of ion flow diagram of *S. baicalensis* extracted by benzene-alcohol method.

We performed GC-MS to extract the volatile components of *S. baicalensis*, and the ion flow results are shown in Figure 4 (In Figure 4A, the peak value of 36.616 is Wogonin, In Figure 4B, the peak values of 35.553 and 36.629 indicate Wogonin, In Figure 4C, the peak value of 35.726 is Wogonin). The TCMSP database was used to identify the volatile components, which are listed in Table 2. The effective components of *S. baicalensis* were also screened by OB and DL screening conditions. The peak area and the percentage of the peak area of GC-MS detection results were used as parameters and the 31 active components were recalculated. The proportion of Wogonin in the total 31 effective volatile components was 49.38%. Wogonin was thus selected for subsequent study.

**TABLE 2:** Volatile components of *S. baicalensis*.

Molecule name	CAS	OB (%)	DL
cis-5,8,11,14,17-Eicosapentaenoic acid	10417-94-4	45.66	0.21
1-Heptatriacotanol	105794-58-9	9.83	0.39
Hexadecanoic acid, methyl ester	112-39-0	18.09	0.12
.delta.-Tocopherol	119-13-1	16.36	0.48
3-tert-Butyl-4-hydroxyanisole	121-00-6	66.46	0.05
Ginsenoyne E	126146-63-2	36.53	0.13
Tributyl phosphate	126-73-8	27.76	0.06
1-Penten-3-one	1629-58-9	69.46	0
Dihydrooroxylin A	18956-18-8	38.72	0.23
Lactose	63-42-3	1.43	0.2
4-Mercaptophenol	637-89-8	60.34	0.01
5-Hydroxymethylfurfural	67-47-0	45.07	0.02
Panaxydol	72800-72-7	61.67	0.13
Cedrol	77-53-2	16.23	0.12
2-Methoxy-4-vinylphenol	7786-61-0	38.39	0.03
.beta.-Guaiene	88-84-6	19.91	0.07
4-Cyclopentene-1,3-dione	930-60-9	49.25	0.01
Benzoic acid, ethyl ester	93-89-0	27.58	0.03
Acetophenone	98-86-2	48.19	0.02
4H-Pyran-4-one, 2,3-dihydro-3,5-dihydroxy-6-methyl-	28564-83-2	37.8	0.03
1H-3a,7-Methanoazulene, 2,3,4,7,8,8a-hexahydro-3,6,8,8-tetramethyl-, [3R-(3.alpha.,3a.beta.,7.beta.,8a.alpha.)]-	469-61-4	55.56	0.1
3-Furaldehyde	498-60-2	50.96	0.01
Orcinol	504-15-4	48.14	0.02
Di-epi-.alpha.-cedrene	50894-66-1	52.87	0.1
(1S,2S)-(+)-N-Methylpseudoephedrine	51018-28-1	37.12	0.04
n-Hexadecanoic acid	57-10-3	19.3	0.1
2,5-Octadecadiynoic acid, methyl ester	57156-91-9	6.88	0.17
9,12-Octadecadienoic acid (Z,Z)-	60-33-3	41.9	0.14
2-Furancarboxaldehyde, 5-methyl-	620-02-0	43.92	0.01
Hexadecanoic acid, ethyl ester	628-97-7	18.99	0.14
Wogonin	632-85-9	30.68	0.23

#### 3.5.2 Prediction of the Targets of Wogonin in Lung Cancer

**FIGURE 5:**
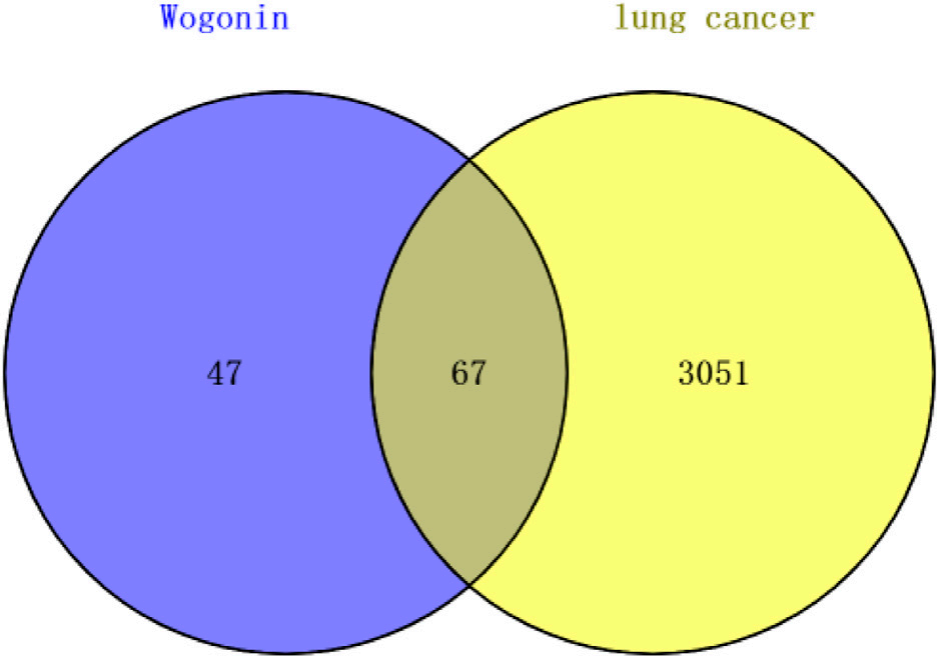
Analysis of common genes among Wogonin targets and lung cancer–related genes.

The targets of Wogonin were predicted using SwissTargetPrediction and BATMAN-TCM databases, and the results identified 114 potential targets. We also used GeneCards and NCBI-Gene databases to investigate potential disease-related genes in lung cancer, and 3,118 genes related to lung cancer were retrieved. Venn diagram results, shown in Figure 5, revealed 67 common genes between the Wogonin targets and lung cancer–related genes.

#### 3.5.3 GO and KEGG Enrichment Analysis

**FIGURE 6:**
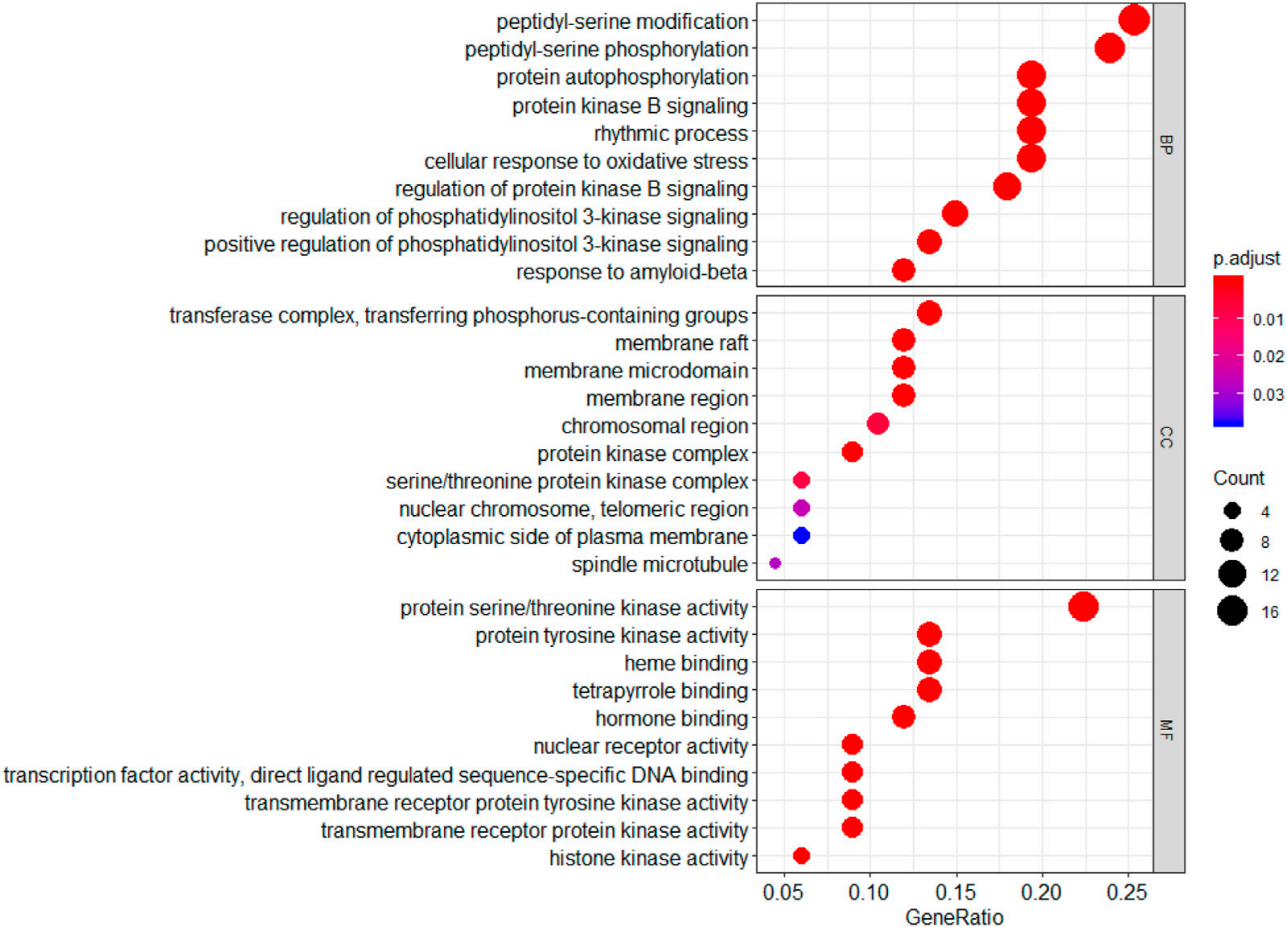
GO annotation of Wogonin targets related to lung cancer.

GO functional annotation analysis revealed that the 67 common genes were involved in biological processes involving peptidye-serine modification and regulation of protein kinase B signaling; cell components including the membrane raft and chromosomal region; and molecular function involving protein serine/threonine kinase activity and heme binding (Figure 6).

**FIGURE 7:**
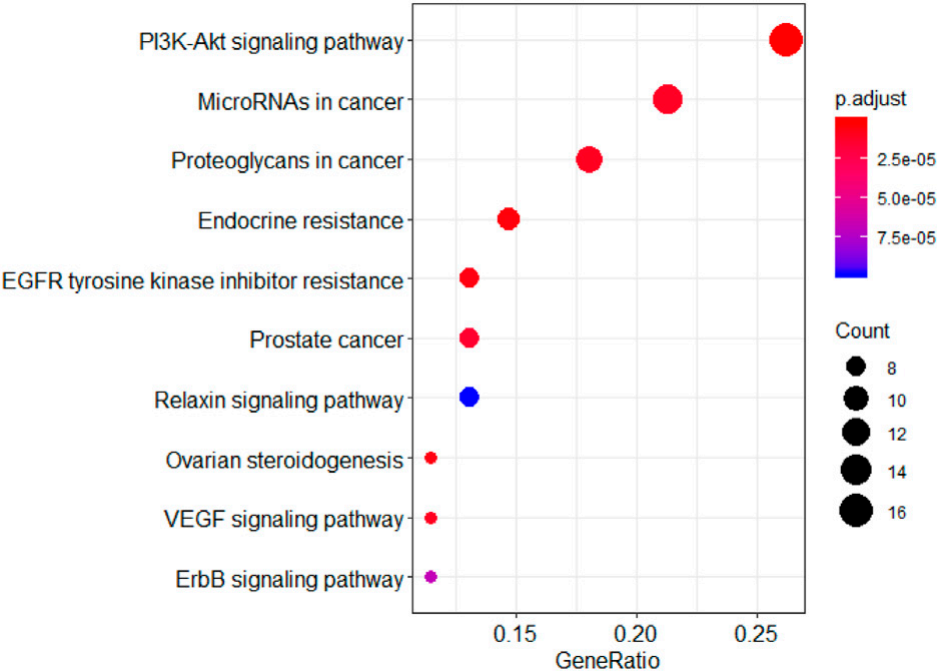
KEGG pathway analysis of Wogonin targets related to lung cancer.

KEGG analysis showed that the genes were involved in pathways such as the PI3K-Akt signaling pathway, ERBB signaling pathway, and EGFR tyrosine kinase inhibitor resistance (Figure 7).

#### 3.5.4 PPI Analysis

**FIGURE 8:**
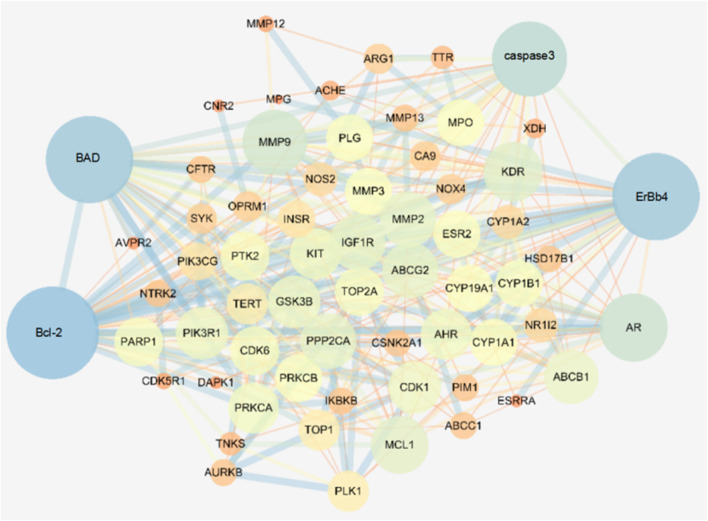
Target interaction regulatory network of Wogonin in lung cancer.

The STRING database was used to retrieve the interaction network of Wogonin on lung cancer target genes, and the non-interacting proteins were deleted. The data were imported into Cytoscape software for network analysis, and the results are shown in Figure 8. Each dot represents a single gene; a larger dot indicates a greater degree of explanation, indicating that the gene is more important in the network. From these results, we selected the apoptotic factor Bad, Bcl-2, apoptotic initiation factor caspase-3/cleaved caspase-3, and ErbB4 protein for subsequent experimental verification.

### 3.6 Wogonin Participates in the Regulation of Proteins and Factors in Lung Cancer

**FIGURE 9:**
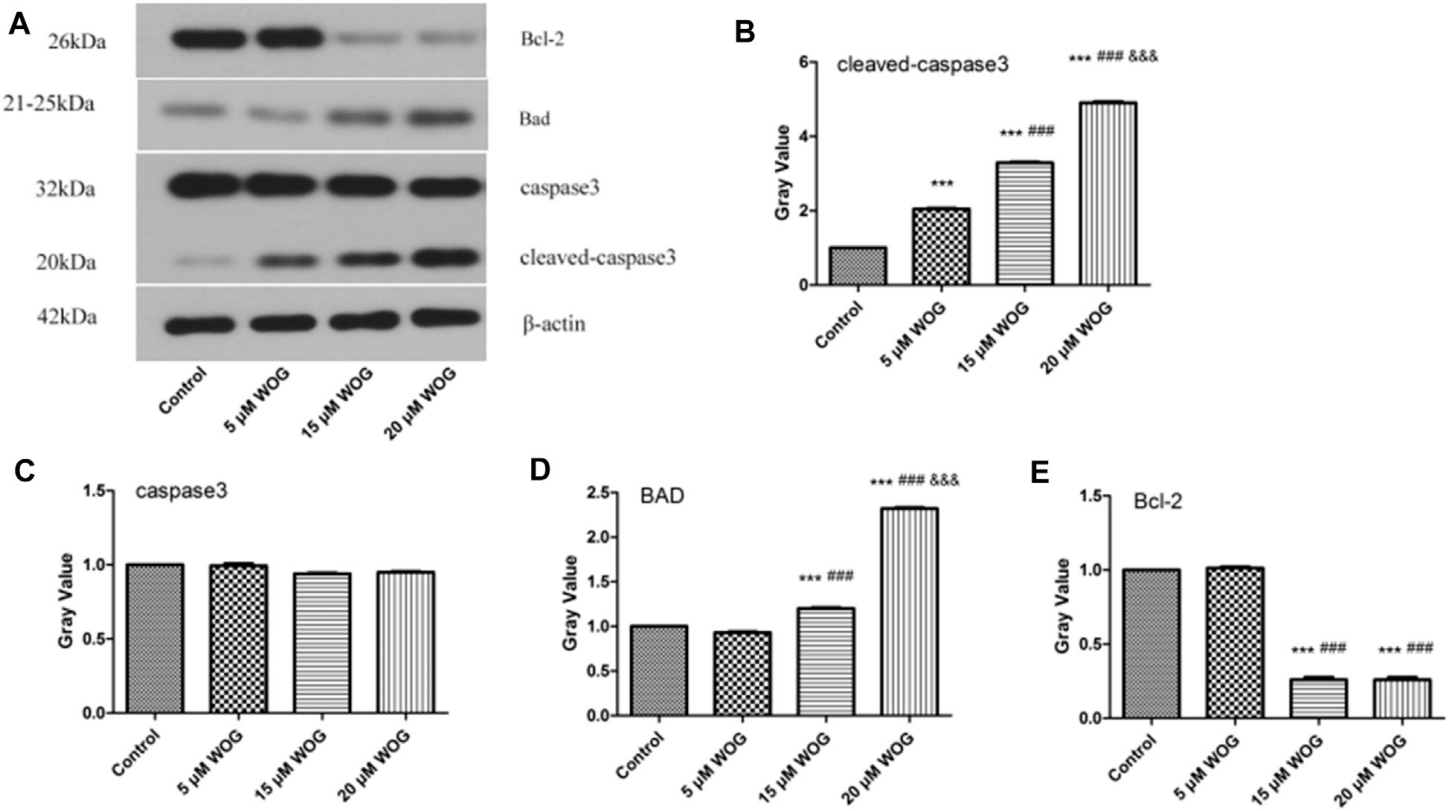
Wogonin regulates the expression of Cleaved caspase-3**(B)**, caspase-3**(C)**, BAD**(D)**, Bcl-2**(E)** in lung cancer cells. compared with the control group, *p < 0.05, **p < 0.01, ***p < 0.001; compared with the 5 mM Wogonin (WOG) group, ^#^ *p* < 0.05, ^##^ *p* < 0.01, ^###^ *p* < 0.001. compared with the 15 mM WOG group, ^&^ *p* < 0.05, ^&&^ *p* < 0.01, ^&&&^ *p* < 0.001.

We performed western blot analysis to evaluate the expressions of key apoptotic factors Bad, Bcl-2, and caspase-3/cleaved caspase-3 in lung cancer cells treated with Wogonin. As shown in Figure 9 and Table 3, the expression levels of cleaved caspase-3 and Bad proteins were significantly higher in lung cancer cells treated with Wogonin compared with control cells (*p* < 0.05). The level of Bcl-2 protein significantly decreased after Wogonin treatment (*p* < 0.05). All changes in protein expression showed dose-dependent responses to Wogonin treatment.

**TABLE 3:** Gray value of protein.

Name	Gray value			
	Control	5 μM WOG	15 μM WOG	20 μM WOG
Cleaved caspase-3	1.00	0.99	0.94	0.95
caspase-3	1.00	0.99	0.94	0.95
Bad	1.00	0.93	1.20	2.32
Bcl-2	1.00	1.01	0.26	0.26

**FIGURE 10:**
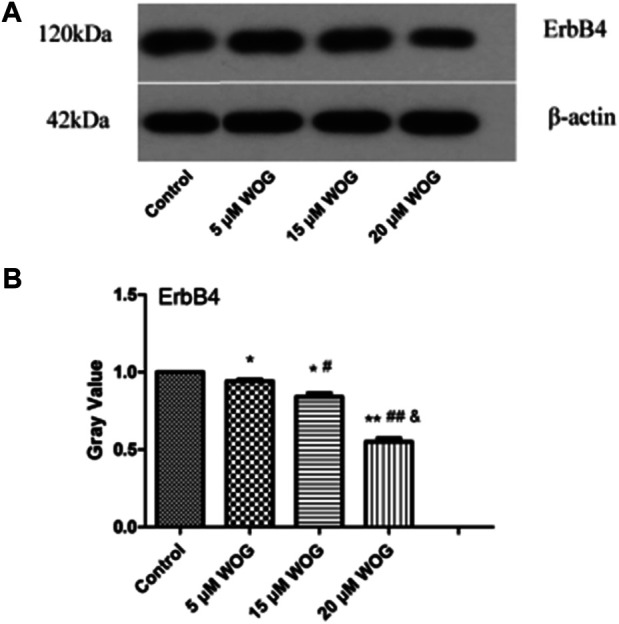
Wogonin induces the decrease of ErbB4 protein in lung cancer cells. **(A)** Gel result, **(B)** Gray value analysis. *p < 0.05, **p < 0.01, ***p < 0.001 compared with the control group. compared with the control group, *p < 0.05, **p < 0.01; compared with the 5 mM Wogonin (WOG) group, ^#^ *p* < 0.05, ^##^ *p* < 0.01. compared with the 15 mM WOG group, ^&^ *p* < 0.05.

We also examined ErbB4, which functions in the ERBB signaling pathway. As shown in Figure 10 and Table 3, ErbB4 expression in lung cancer cells was significantly decreased upon treatment with Wogonin in a dose-dependent manner (*p* < 0.05).

### 3.7 The Regulatory Pathway of Wogonin in Lung Cancer Cells

**FIGURE 11:**
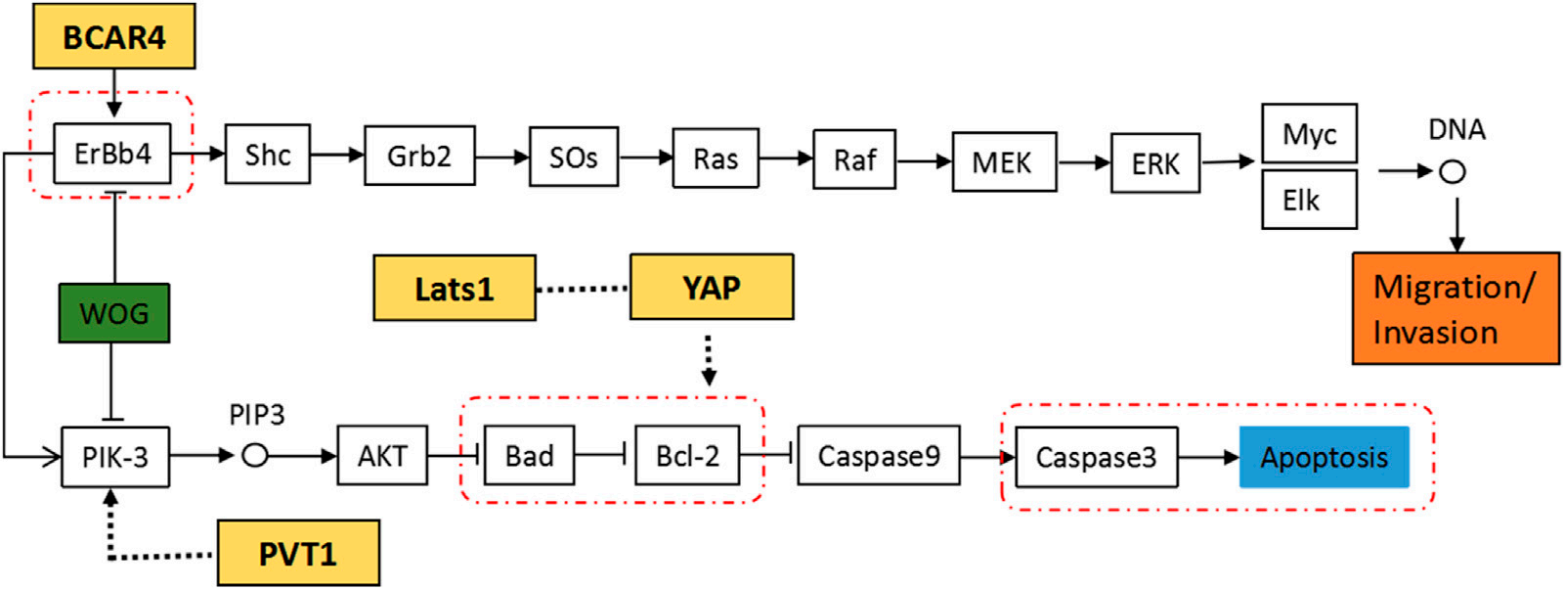
Regulatory network of Wogonin in apoptosis in lung cancer.

Combining the susceptibility genes associated with serological indicators, KEGG enrichment results and experimental verification results, we constructed the regulatory pathway of Wogonin in lung cancer cells, as shown in Figure 11. Our results suggest that Wogonin may induce the apoptosis of lung cancer cells by inhibiting the PIK-3 signaling pathway, inducing the expression of the pro-apoptotic factor Bad and activating cleaved caspase-3, as well as inhibiting the expression of the anti-apoptotic factor Bcl-2. Wogonin may also inhibit the expression of ErbB4, thus affecting the downstream signaling pathway and inhibiting the invasion and metastasis of lung cancer cells. In addition, the lung cancer serological indicator NSE susceptibility gene product LATS1 may interfere with apoptosis of tumor cells by regulating the CYFRA21-1 susceptibility gene product YAP. The CA153 susceptibility gene product BCAR4 may interfere with the ERBB4 signaling pathway, and the CA125 susceptibility gene product PVT1 may participate in the regulation of the PIK-3 signaling pathway.

## 4 Discussion

Traditional doctor in the clinical diagnosis, often through judging tumor form to do, there is strong subjectivity, In addition, the medical expertise varies among doctors, and misdiagnosis can sometimes occur. Doctors need to deal with extremely large amounts of data, artificial problems such as medical resources stress, low efficiency. The same kind of treatment method is often used in many diseases cannot achieve targeted, individual treatment.

The continuous development of technology has enabled researchers to conduct comprehensive and accurate analyses of tumors. An increasing number of studies have applied artificial intelligence algorithms to the field of tumor diagnosis (Pang et al., 2019; Yang et al., 2019). Mining medical data may improve the efficiency of disease diagnosis and reduce the cost of disease discovery. How to establish an effective and accurate tumor diagnosis model to assist doctors in clinical diagnosis and treatment is a key problem and one of the focuses of this study. Several artificial intelligence algorithm models are widely used, including the BP Neural Network model, SVM model, and Linear Regression model. In recent years, research has been focused on SVM, which is an excellent classifier with strong generalization ability and relatively high accuracy (Liu et al., 2014). Zhang et al. used a comprehensive GEO database and SVM to detect breast cancer biomarkers to distinguish between normal samples and cancer samples; the discriminant model using cross validation to the accuracy, at the same time using cross-validation score as evaluation standard, and the SVM algorithm and other algorithms are compared. The recursive SVM algorithm with the highest classification accuracy was obtained, and 15 biomarkers of breast cancer were found. Yousef et al. applied an algorithm combining a SVM algorithm and gene expression network to select tumor biomarkers and classify tumors (Yousef et al., 2009). Multiple gene expression networks were input into the SVM classification model for training; the accuracy of the results was fed back to the model, and the gene expression networks were constantly cross-exchanged. Until you choose the model with the highest classification accuracy.

NSE, CYFRA21-1, CA125 and CA153 are commonly used serum indicators for the clinical diagnosis of lung cancer. In this study, combined with the four serological indicators, the SVM parameters were optimized by the grid search method for modeling. The optimal parameters were found by grid search method as follows: *c* = 90.597, and *g* = 32. Under these parameters, the accuracy of the test set was 90.8333%, and the number of false positives and false negatives was 9 and 2, respectively; c, g step size is 0.5. In this study, we successfully constructed an SVM model based on tumor markers that can be used as a reference for the auxiliary diagnosis of lung cancer. Susceptibility genes can regulate the expression level of serological indicators in the body, and the expression level of serological indicators can also influence or reflect the related genes. The expression levels of NSE, CYFRA21-1, CA125 and CA153 in patient serum may have a certain regulatory effect on key genes of lung cancer, and this requires further study. We obtained susceptibility genes corresponding to the four serological indexes associated with lung cancer as follows: NSE, *LATS1*; CA153, *BCAR4*; CYFRA21-1, *YAP*; and CA125, *PVT1*.

Chinese herbal medicine, with a variety of targets and low side effects, is a promising treatment strategy for lung cancer. However, the ingredients of Chinese herbal medicine are complex. In addition, the study of the mechanism of action is challenging because of the many pharmacological effects of Chinese herbal medicines and the extensive time required for animal studies. Therefore, network pharmacologic approaches are very useful because unlike traditional drug research, they have the ability to address multiple proteins or networks involved in drug-targeted diseases (Malik et al., 2017). Network pharmacology has previously analyzed the mechanism of action of Chinese herbal compounds or a single Chinese herbal medicine on tumors (Pacheco et al., 2019). However, few reports have used network pharmacology to explore the potential effects of volatile extractives from Chinese herbal medicine on tumors. Poornima P. et al. examined the mechanism of β-elemene (a volatile oil component in Chinese herbal medicines such as ginseng) against peritoneal effuse in patients with advanced pancreatic cancer using a network pharmacology method and performed preliminary verification using molecular experiments (Poornima et al., 2016). This study established a role for network pharmacology research in exploring the constituents of volatile oil from Chinese herbal medicine.

A variety of chemical components, including flavonoids, phenolic acids, amino acids, sterols, essential oils, and trace elements, have been discovered in *S. baicalensis* (Wang Z. L. et al., 2018). *S. baicalensis* contains rich volatile oil, which has antibacterial, anti-inflammatory and anti-tumor activities (Junqiu et al., 2019). To further analyze the volatile extractives of *S. baicalensis* and the mechanisms of action against lung cancer, we examined the volatile extractives of *S. baicalensis* and screened the components related to lung cancer. Our findings identified Wogonin as a volatile component of *S. baicalensis* that is associated with lung cancer. Studies have shown that Wogonin has cytotoxic effects on lung cancer cells and is associated with the activation of apoptosis and the production of reactive oxygen species (Pant et al., 2012). Another study showed that by inducing TIMP-2 expression and decreasing TGF-1, Wogonin inhibited the activity of MMP-2, APN and EGFR-TPK, activated caspase 3, and played an important role in the metastasis and apoptosis of lung cancer A549 cells (Chengyang and Chuangcheng, 2019). Using network pharmacology, we analyzed the potential targets and molecular mechanisms of Wogonin in lung cancer. Functional enrichment and pathway enrichment analyses were performed on the 67 common genes retrieved from four databases using KEGG and GO analysis. The results showed that Wogonin may act on lung cancer through inhibition of EGFR tyrosine kinase by the PI3K-Akt signaling pathway and the ERBB signaling pathway.

Further analysis revealed that the targets of Wogonin were distributed in the PI3K-Akt signaling pathway, which is an important pathway involved in tumor cell proliferation (Qu et al., 2017; Curigliano and Shah, 2019). The Bcl-2 protein is the core regulator of mitochondrial apoptosis and a recognized anticancer target (Alzahrani, 2019). The downstream factor Bad regulates cell apoptosis and survival and is a pro-apoptotic factor. One study showed that targeting anti-apoptotic Bcl-2 protein kills lung cancer cells by activating the mitochondrial cell death program (Sun et al., 2017). Our study showed that Wogonin inhibited the expression of Bcl-2 and promoted the expression of Bad and the activation of caspase-3/cleaved caspase-3, thereby inducing the apoptosis of lung cancer cells.

The EGFR family is the most studied receptor protein-tyrosine kinase group and has an important role in signal transduction and tumorigenesis (Akane et al., 2017). The family comprises ERBB1–4 (Liu et al., 2017), and ERBB4 is unique because it is the only member that has growth-inhibiting properties (Robert, 2019). In recent years, information on the role of ERBB4 in many tumors has emerged, and most studies have focused on the tumor suppressor function of ERBB4 (Segers et al., 2020). However, there are some conflicting studies that merit re-examination of this conclusion. Our results showed that Wogonin resulted in reduced expression of ERBB4, thus preventing the invasion and metastasis of ERBB4-mediated lung cancer cells.

Combined with the serological index susceptibility genes of lung cancer queried above, we found them through literature search, Studies have shown that lncRNA PVT1 (the CA125 susceptibility gene) is involved in the regulation of immune checkpoints in a variety of tumors, including ovarian cancer, thereby promoting tumor progression (Mattick and Lee, 2009; Chen et al., 2018). Up-regulation of BCAR4 (the CA153 susceptibility gene) in the human breast cancer ZR-75-1 cell line promotes the phosphorylation of ERBB2 and ERBB3, enhances the estrogen-dependent effect of tumor cells, and stimulates the proliferation of tumor cells (Ge and Wang, 2017). LATS1 (the NSE susceptibility gene) is a member of the DBF2-related nucleoprotein kinase family, and together with MST1 constitutes a pathway in the Hippo signaling pathway that inhibits excessive cell proliferation. *In vitro* experiments showed that overexpression of LATS1 inhibits the expression of YAP (the CYFRA21-1 susceptibility gene), thereby inducing G1 phase arrest, inhibiting cell proliferation and promoting tumor cell apoptosis (Luo et al., 2014). We constructed the signal pathway by which Wogonin regulates key targets in lung cancer, including the serum index susceptibility genes. The expression level of lung cancer serological indicators was previously associated with the key targets of lung cancer, which may be used for prognosis judgment in the treatment of lung cancer. Further in-depth studies are needed.

In conclusion, we used an optimized SVM algorithm to construct a serology-based lung cancer diagnosis model and explored volatile constituents in *S. baicalensis* as potential treatments for lung cancer. We comprehensively analyzed the potential targets and molecular mechanisms of Wogonin in regulating lung cancer through network pharmacology. The validity of our network pharmacology analysis was confirmed by *in vitro* studies investigating the key targets. In addition, in combination with the susceptibility genes of serological indicators, the signaling pathway of Wogonin regulating lung cancer was constructed. Our results provide a promising approach to uncover the scientific basis and therapeutic mechanism of Chinese herbal medicine in disease treatment.

## Data Availability

The datasets presented in this article are not readily available because The data is part of the overall project and cannot be disclosed. Requests to access the datasets should be directed to jZ,petermails@zzu.edu.cn.
